# Research trends and hotspots of neurodegenerative diseases employing network pharmacology: A bibliometric analysis

**DOI:** 10.3389/fphar.2022.1109400

**Published:** 2023-01-12

**Authors:** Jie Zhu, Qingchun Liang, Siyi He, Chen Wang, Xiafei Lin, Duozhi Wu, Guanwen Lin, Zhihua Wang

**Affiliations:** ^1^ Department of Anesthesiology, Hainan General Hospital, Hainan Affiliated Hospital of Hainan Medical University, Haikou, Hainan, China; ^2^ Department of Anesthesiology, The Third Affiliated Hospital, Southern Medical University, Guangzhou, China

**Keywords:** network pharmacology, neurodegenerative diseases, Alzheimer’s disease, traditional Chinese medicine, Citespace, VOSviewer, bibliometrics

## Abstract

**Background:** Employing network pharmacology in neurodegenerative diseases (NDs) has been extensively studied recently. However, no comprehensive study has conducted on this subject employing bibliometrics so far. The purpose of this study was to find out the developmental trends and hotspots, and to predict potential research directions in this filed.

**Methods:** Relevant research were collected from the Web of Science Core Collection Bibliometrics and visual analysis were executed using CiteSpace, VOSviewer, Histcite and R-bibliometrix.

**Results:** A total of 420 English articles on network pharmacology in NDs published in 2008–2022 were obtained from the WOSCC database. From 2008 to 2022, annual publications showed a steady growing trend, especially in 2014–2022. China, Beijing Univ Chinese Med, Frontiers in Pharmacology, and Geerts H are the most prolific country, institution, journal, and author, respectively. China, Nucleic Acids Research, and Hopkins AL are the most highly cited country, journal, and author, respectively. Moreover, network pharmacology and Alzheimer’s disease are the focal areas of current researches according to analysis of co-cited references and keywords. Finally, in the detection of burst keywords, systems pharmacology and database are new approaches to disease and drug research, while traditional Chinese medicine (TCM) and Alzheimer’s disease are hot research directions. The above keywords are speculated to be the research frontiers.

**Conclusion:** Network pharmacology and Alzheimers’ disease are the main topics of researches on network pharmacology in NDs. Network pharmacology and the TCM treatment of Alzheimer’s disease have been the recent research hotspots. To sum up, the potential for exploring TCM treatment of AD with network pharmacology is huge.

## Introduction

Neurodegenerative diseases (NDs) refers to a range of brain and spinal cord diseases caused by progressive neuronal damage (MT, 2016). It is characterized by disease-specific protein misfolding, accumulation, and aggregation etc (Dugger and Dickson, 2017). It includes two main types: one primarily affects memory and cognitive function, such as Alzheimer’s disease (AD), the other chiefly influences motor function, such as Parkinson disease (PD) (Solanki et al., 2016). In recent years, the incidence of neurodegenerative disease has been increasing (Hou et al., 2019). AD is the most common neurodegenerative disease at present, followed by PD (GBD 2016 Dementia Collaborators, 2019). However, these diseases can not be completely cured (The Lancet, 2016), in fact can only be improved by alleviating associated symptoms (Breijyeh and Karaman, 2020). Therefore, further researches on the treatment of NDs may be the general trend. A number of Chinese herbal medicines with antioxidant and anti-inflammatory properties have been proved to be beneficial in the treatment of NDs (Li et al., 2014).

Traditional Chinese medicine (TCM) is marked by its complex components, multiple targets and complicated pathways of activation (Wang et al., 2018). Traditionally, it has been difficult to explain how TCM formulae work in terms of pharmacodynamics and rules of action. Network pharmacology integrates high-throughput data integration, target conjecture, information mining and database retrieval etc (He et al., 2022). The emergence of network pharmacology depends on the expeditious development of systems biology and network technology. Based on the interaction network of drugs, diseases and targets, the intervention and influence of drugs on the body’s disease network can be completely understood, and the discovery of new drugs and new targets can be guided (Nogales et al., 2022). As a new paradigm to guide drug development and application, network pharmacology now is widely used in the exploration of many diseases and drugs (Lai et al., 2020). The holistic and systematic view of network pharmacology coincides with TCM, and plays a critical role in exploring the targets, understanding the biological basis of diseases and syndromes, and illuminating the regulatory mechanism of traditional Chinese herbal medicines (Wang et al., 2021). For example, sildenafil was identified as a potential Alzheimer’s disease drug candidate through network medicine strategy (Fang et al., 2021) recently. Nevertheless, there are no studies systematically analyzed and visualized the researches on the network pharmacology in NDs.

In contrast to traditional reviews, bibliometrics analyzes published articles information and related data, applying statistical methods to describe or visualize the relationship between articles (Ninkov et al., 2022). Bibliometrics has been widely used to track the evolution and development of knowledge fields as well as to identify potential research hotspots (Chen, 2004). Through the visual display of bibliometrics, researcher can identify the development of a certain field more intuitively, comprehensively and systematically (Chen et al., 2020). At present, no bibliometric research has conducted on the network pharmacology in NDs. Therefore, our study aims to show evolutionary trends and emerging hotspots of researches on network pharmacology in NDs, provide guidance for further research direction and scientific decision-making based on the WoSCC database by using visual tools.

## Methods

### Data sources and search strategies

**FIGURE 1:**
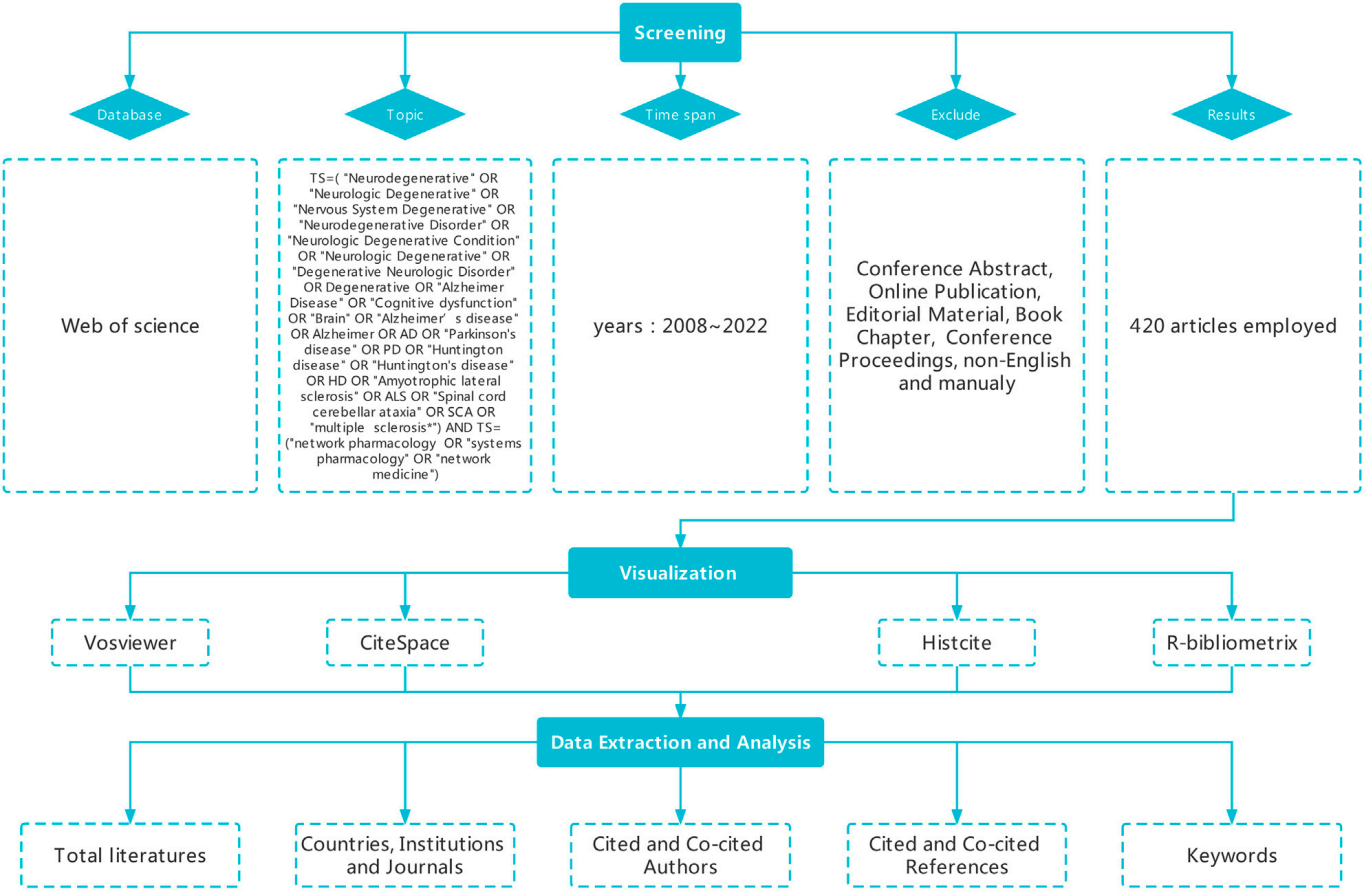
Flow chart of literature retrieval.

Relevant data was searched and obtained from the WoSCC in 2008–2022. Literature retrieval was conducted within a day (4 September 2022) to avoid fluctuations in rapid updates of publications. The search formula was constructed to TS= (“Neurodegenerative” OR “Neurologic Degenerative” OR “Nervous System Degenerative” OR “Neurodegenerative Disorder” OR “Neurologic Degenerative Condition” OR “Neurologic Degenerative” OR “Degenerative Neurologic Disorder” OR Degenerative OR “Alzheimer Disease” OR “Cognitive dysfunction” OR “Brain” OR “Alzheimer’s disease” OR Alzheimer OR AD OR “Parkinson’s disease” OR PD OR “Huntington disease” OR “Huntington’s disease” OR HD OR “Amyotrophic lateral sclerosis” OR ALS OR “Spinal cord cerebellar ataxia” OR SCA OR “multiple sclerosis*“) AND TS= (“network pharmacology” OR “systems pharmacology” OR “network medicine”). A total of 752 articles were retrieved, the main types of literatures chosen for our study were articles and reviews, and the language was confined to English. Two researchers (ZJ and HSY) independently performed the original literatures search and identify any possible errors and discrepancies. Besides, they read the titles, abstracts, and even the full texts of paper to filter out papers that are closely related to our subject. Only 420 publications were enrolled. The detailed retrieval process was shown in Figure 1.

### Statistical analysis

In this study, visual analysis was constructed using Microsoft Office Excel 2019, HistCite (version 2009.08.24), VOSviewer (version1.6.18), CiteSpace (version6.1), and Bibliometrix 4.1.0 Packages based on the R language.

Microsoft Excel was employed to make the tables and display the information of countries, authors, institutions and references.

HistCite, a document indexing and analysis software designed to process information from literature searches, helps scholars master the historical development of a field and identifies highly cited research and scholars (Garfield et al., 2006). In present study, it was used in calculating the publications, reference citation, total local citation score (TLCS) and total global citation score (TGCS). TLCS refers to total citations in the collection, which can reveal the popularity of the article in this field and is usually less than TGCS. And TGCS refers to total citations in Web of Science to papers in the collection. Although TGCS does not directly reveal the focus of the field, it can still help to find the most influential work in all disciplines.

Vosviewer, a software for building a visual bibliometric network based on network data (van Eck and Waltman, 2010), is hired to construct visual network atlas for systematically understanding the structure and dynamic development of scientific researches. In this study, VOSviewer 1.6.18 was used to visualize the cooperative networks of productive countries, journals and authors based on bibliographic data.

CiteSpace is a citation analysis software based on scientometrics, data, and information visualization to visualize the distribution, patterns, and relationships of scientific knowledge (Chen, 2004). It can build visual networks, compute intermediary centrality, and perform burst detection to reveal changes in emerging trends, identify research frontiers (Chen et al., 2012), and tag key keywords (Chen, 2006). The visualization map is composed of nodes and lines (Chen and Song, 2019). Nodes are sized based on the number of items, and connections between nodes indicate co-occurrences, collaborations, or citations. The betweenness centrality measure quantifies the importance and connectivity of a node’s position within the network. The higher the betweenness centrality it is, the more connections it passes through (Freeman, 1978). Circles with a betweenness centrality over 0.1 were highlighted in purple. In clustering, the modularity Q and mean silhouette value are the most important parameters. Clusters were considered dominant and persuasive when Q > 0.3 and mean silhouette value >0.5 (Xu et al., 2022). In present study, CiteSpace was employed to analyze co-cited authors and documents. CiteSpace can also identify burst citations and keywords. Basic parameters of CiteSpace were set according to our previous study (Dong et al., 2021).

The bibliometrix package in R 4.1.0 was used to automate the transformation and analysis of bibliographic information for selected publications, including institutional and periodical outputs, as well as author impact indicators. Indicators including number of publications and number of citations were used to evaluate the impact factors of the authors. The h-index commonly used to assess the level of academic output of a researcher (Bertoli-Barsotti and Lando, 2017), higher h-indix indicates higher scholarly impact (Hirsch, 2005). As a derivative index of h-index, g-index can further measure the academic impact and achievements of scholars (Ali, 2021).

## Results

### Analysis of publication outputs and citation

**FIGURE 2:**
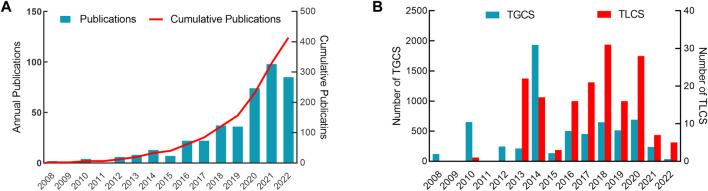
Overall distribution of publication outputs and citations on network pharmacology in NDs. **(A)** Number and trends of annual and cumulative publications. **(B)** Annual number of TLCS and TGCS.

A total of 420 English articles related to the network pharmacology in NDs were included. Figure 2A revealed an overall growing trend for annual and cumulative quantity of publications released from 2008 to 2022. Figure 2B showed that annual number of TGCS peaked in 2014 (*n* = 1934), and TLCS peaked in 2018 (*n* = 31). 2014–2022 was a period of stable development in this field with 394 publications. However, TGCS and TLCS fluctuated during this period. The above results showed that although the number of citations fluctuated, the overall volume continued to grow sharply.

### Distribution of countries/regions

**FIGURE 3:**
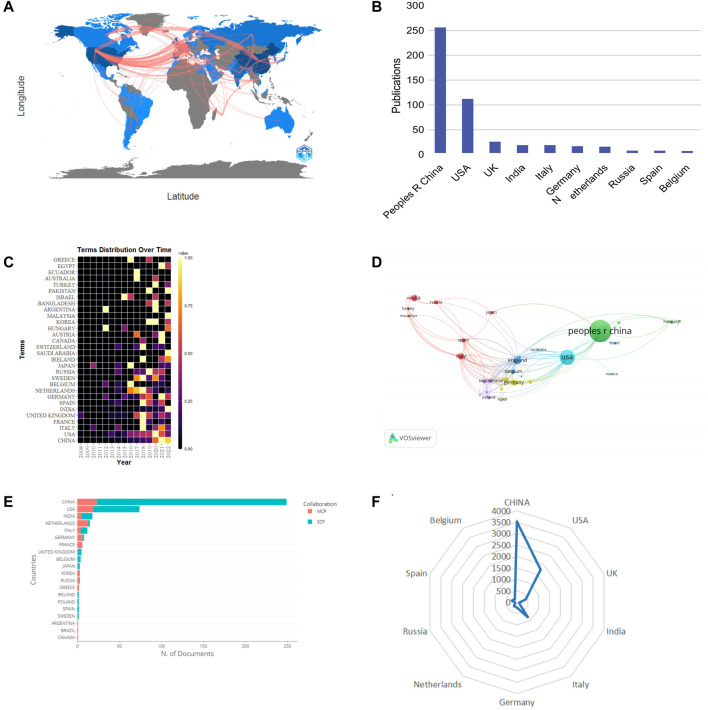
Analysis of countries/regions. **(A)** World map of the intensity of cooperation between countries. **(B)** Publications of the top 10 countries. **(C)** Distribution of the countries/regions’ publications over time. **(D)** Cooperation network of countries. **(E)** Distribution of corresponding author’s country. MCP refers to the number of papers co-authored with different countries, and SCP refers to the number of papers co-authored with same countries. **(F)** Radar map of TGCS of the top 10 productive countries.

From 2008 to 2022, a total of 53 countries participated in the research on network pharmacology in NDs. The United States had close connections with European countries (Figure 3A). China was the most prolific country (256 publications), followed by the United States (109 publications) and the UK (26 publications) (Figure 3B). There was a rising trend in the number of publications in the main distribution countries (Figure 3C). In Figure 3D, nodes are sized according to the number of publications, the lines indicate the cooperation, and thickness of line represents the strength of connections. China had the highest quantity of publications, while the United States had more links between other countries. Additionally, The US, Italy and China had the highest centrality over 0.1 (Table 1), indicating that these countries were the major research centers in the area of the network pharmacology in NDs. According to Figure 3E, although China had a great number of articles, it had a low proportion of co-operation with authors from other countries. The most cited countries were China (3,525), followed by United States (1,760) and Italy (801) (Figure 3F). The results mentioned above pointed out that China and the United States had significant contributions and influence in this field.

**TABLE 1:** Top 10 countries by publications, citations and centrality.

Rank	Country	Publications	TGCS^a^	Average citations	Rank	Country	Centrality
1	China	256	3,525	13.77	1	United States of America	0.53
2	United States of America	109	1,760	16.15	2	Italy	0.38
3	United Kingdom	26	406	15.62	3	China	0.2
4	India	19	98	5.16	4	United Kingdom	0.17
5	Italy	19	801	42.16	5	Spain	0.17
6	Germany	17	237	13.94	6	Ireland	0.14
7	Netherlands	16	215	13.44	7	Turkey	0.14
8	Russia	8	75	9.38	8	Russia	0.11
9	Spain	8	227	28.38	9	Germany	0.09
10	Belgium	7	156	22.29	10	Hungary	0.08

^a^ TGCS, total global citation score.

### Analysis of institutions

**FIGURE 4:**
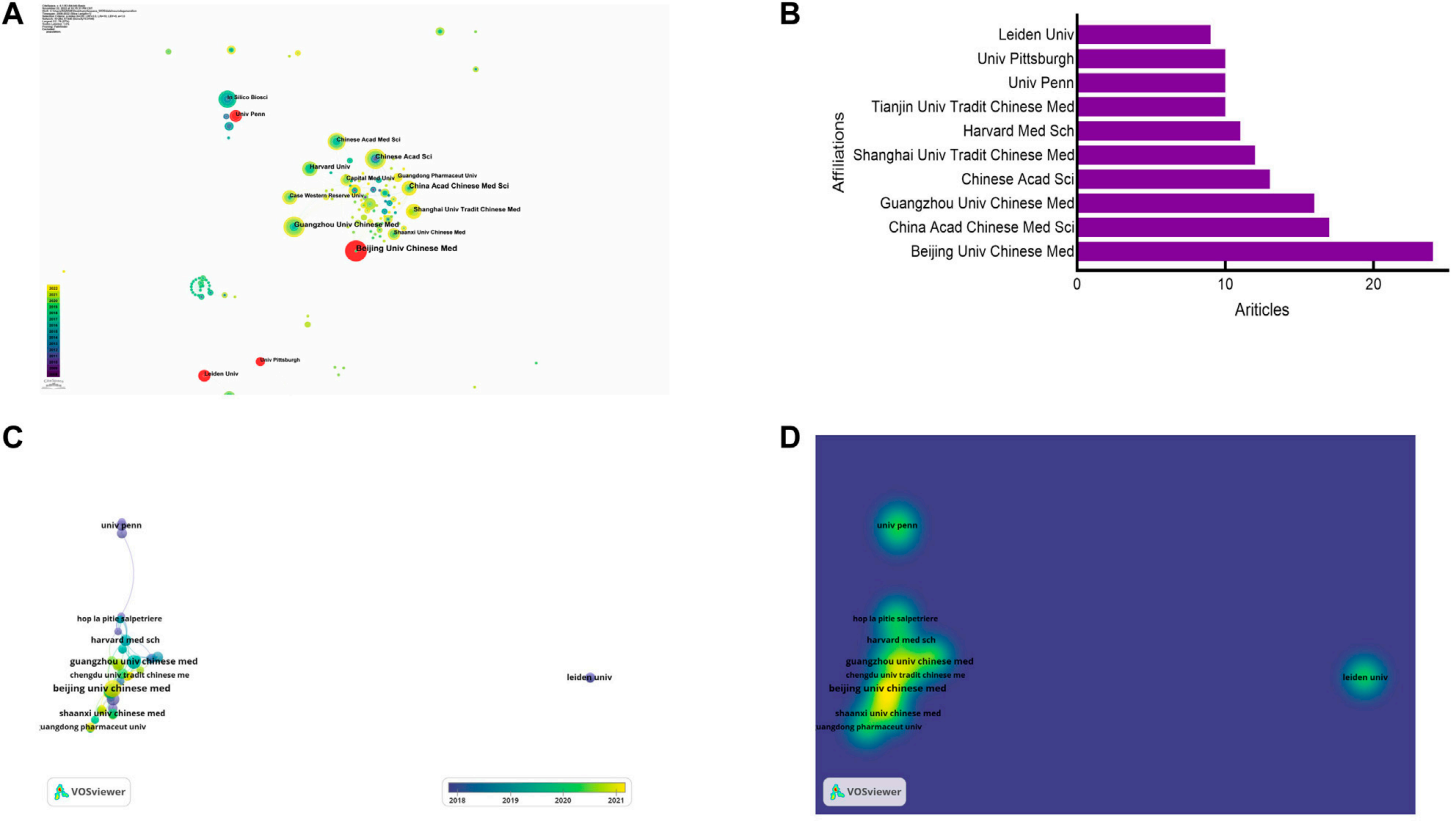
Analysis of institutions. **(A)** Visualization of institutions involved in network pharmacology in NDs. **(B)** Top 10 institutions of publications. **(C)** Cooperation network of institutions. **(D)** Density map of institutions cooperation.

Information visualization helps to identify influential institutions and clarify their cooperation. Chinese institutions are the main driving force in this area and top three institutions ranked by centrality were China Acad Chinese Med Sci (0.06), Guangzhou Univ Chinese Med (0.04) and Beijing Univ Chinese Med (0.03) (Figure 4A; Table 2). However, their centrality were less than 0.1, indicating that there are no key research center and sufficient inter-agency cooperation among the top 10 institutions. Beijing Univ Chinese Med (24 publications), China Acad Chinese Med Sci (17 publications), Guangzhou Univ Chinese Med (16 publications) were the top three highly productive institutions in this field (Figure 4B; Table 2). Although Chinese Acad Sci was not the institution with the largest number of publications, it had the highest TGCS, representing its great influences in all disciplines. In Figures 4C, D, the institutions owning five or more publications were used to create collaboration network. Inter-agency collaboration is considered crucial motivation to conduct large-scale research. We observed that the cooperative relationship between institutions was relatively simple, and correlation was not close. In short, China is the main driving force in this filed, but interagency cooperation still needs to be strengthened.

**TABLE 2:** Top 10 institutions distributed by publications and centrality.

Rank	Institution	Publications	TGCS^a^	Original country	Institution	Centrality	Original country
1	Beijing Univ Chinese Med	24	84	China	China Acad Chinese Med Sci	0.06	China
2	China Acad Chinese Med Sci	17	197	China	Guangzhou Univ Chinese Med	0.04	China
3	Guangzhou Univ Chinese Med	16	248	China	Beijing Univ Chinese Med	0.03	China
4	Chinese Acad Sci	13	1,851	China	Tianjin Univ Tradit Chinese Med	0.03	China
5	Shanghai Univ Tradit Chinese Med	12	69	China	Chinese Acad Sci	0.02	China
6	Harvard Med Sch	11	242	United States of America	Capital Med Univ	0.02	China
7	Tianjin Univ Tradit Chinese Med	10	87	China	Shaanxi Univ Chinese Med	0.01	China
8	Univ Penn	10	255	United States of America	Case Western Reserve Univ	0.01	United States of America
9	Univ Pittsburgh	10	69	United States of America	Guangdong Pharmaceut Univ	0.01	China
10	Leiden Univ	9	82	Netherlands	Fudan Univ	0.01	China

^a^ TGCS, total global citation score.

### Analysis of authors and Co-Cited authors

**FIGURE 5:**
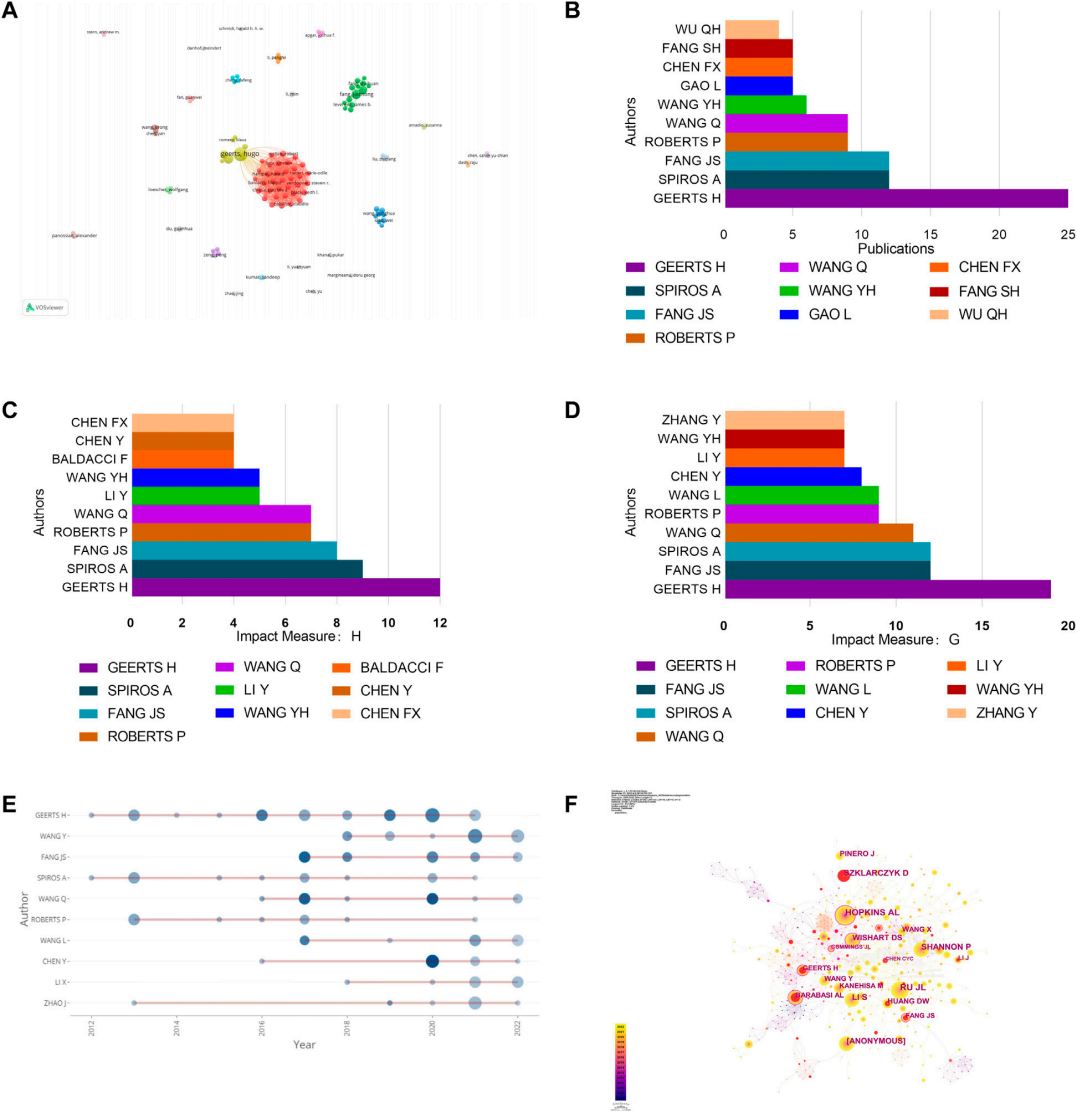
Analysis of authors and co-cited authors. **(A)** Cooperation network of authors, 112 authors had published at least three documents. **(B)** Top 10 authors in terms of number of publications. **(C)** Top 10 authors in terms of H-index **(D)** Top 10 authors in terms of G-index. **(E)** The top 10 authors’ production over time. **(F)** CiteSpace visualization of co-cited authors.

A total of 2,511 authors contributed to researches on network pharmacology in NDs. Geerts H, from the United States, had the largest number of publications (25), followed by Fang JS (12) and Spiros A (12) (Table 3). Moreover, most of the influential authors were from China and the United States. As shown in Figure 5A, Geerts H cooperated closely with Roberts P, Carr R and Spiros A. In addition, Fang JS frequently collaborated with Wang Q. In Figures 5B–D, Geerts H had the largest number of publications, highest H-index and G-index, indicating his outstanding contribution to this field. As early entrants into the field, Geerts H and Spiros A continued to publish related papers from 2012 to 2021, and Geerts H almost had important outputs each year (Figure 5E). As noted in Figure 5F, Hopkins AL had the highest co-citations, followed by Ru JL and Shannon P, with centrality more than 0.1 marked with purple rings, suggesting that they had strong academic influences in this field.

**TABLE 3:** Top 10 authors distributed by publications.

Rank	Author	Publications	Publications as key author^a^	Country	Institution	TGCS^b^	TLCS^c^
1	Geerts H	25	19	United States	Silico Biosciences Perelman School of Medicine	374	45
2	Fang JS	12	10	China	Guangzhou University of Chinese Medicine	210	28
3	Spiros A	12	1	United States	Silico Biosciences Perelman School of Medicine	200	22
4	Wang q	9	8	China	Guangzhou University of Chinese Medicine	236	20
5	Roberts P	9	2	United States	Silico Biosciences Perelman School of Medicine	155	18
6	Wang YH	6	5	China	Northwest A&F University	1,828	6
7	Cheng FX	5	5	United States	Cleveland Clinic	81	4
8	Fang SH	5	2	China	Guangzhou University of Chinese Medicine	112	14
9	Gao L	5	0	China	Shanxi University of Chinese Medicine	125	14
10	Wu QH	4	1	China	Hainan Provincial Hospital of Traditional Chinese Medicine	45	0

^a^ Publications as key author, the number of publications as first author or corresponding author.

^b^ TGCS, total global citation score.

^c^ TLCS, total local citation score.

### Analysis of journals

**FIGURE 6:**
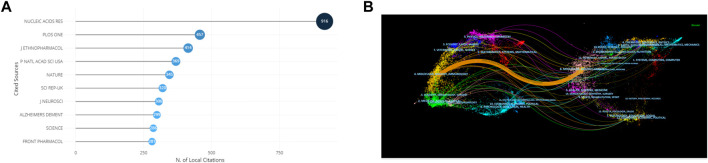
Analysis of journals and co-cited journals. **(A)**Top 10 co-cited journals. **(B)** Dual-map overlay of journals.

All articles are distributed in 193 journals, and four of these journals have more than 10 papers. We derived impact factors (IF) and journal quartiles from the 2021 Journal Citation Reports. The top three high-yield journals were Frontiers in Pharmacology (IF5.988), Evidence-based Complementary and Alternative Medicine (IF2.650), and Journal of Ethnopharmacology (IF5.195). The top 10 most productive journals in Table 4 were mainly ranked in Q1 or Q2, indicating that the above journals had high scholastic reputations in this field. The three most frequently cited journals were Nucleic Acids Research (IF19.160), PLoS One (IF3.752) and Journal of Ethnopharmacology (IF5.195) **(** Figure 6A **)**. The distribution of scientific journal issues is shown in a double map overlay (Figure 6B). The double map overlay of journals displayed one core citation way, the citation journals are mainly distributed in the area of molecular, biology and immunology, and the cited articles published in journals were predominantly contained in the area of molecular, biology and genetics.

**TABLE 4:** Top 10 journals distributed by publications.

Rank	Journal	Publications	% Of 420	IF(JCR 2021)	TGCS	JIF quartile	H-index	G-index
1	Frontiers in pharmacology	39	9.28	5.988	292	Q1	12	16
2	Evidence-based complementary and alternative medicine	33	7.86	2.650	108	Q3	6	9
3	Journal of ethnopharmacology	19	4.52	5.195	334	Q1	10	15
4	International journal of molecular sciences	10	2.38	6.208	170	Q1	7	7
5	CPT-pharmacometrics and systems pharmacology	9	2.38	4.339	82	Q2	5	8
6	Frontiers in aging neuroscience	8	1.90	5.702	53	Q1	3	3
7	Molecules	8	1.90	4.927	67	Q2	5	6
8	Phytomedicine	8	1.90	6.656	41	Q1	4	6
9	Alzheimers and dementia	7	1.67	16.655	179	Q1	5	5
10	Biomedicine and pharmacotherapy	7	1.67	7.419	94	Q1	4	7

### Analysis of cited and co-cited references

**FIGURE 7:**
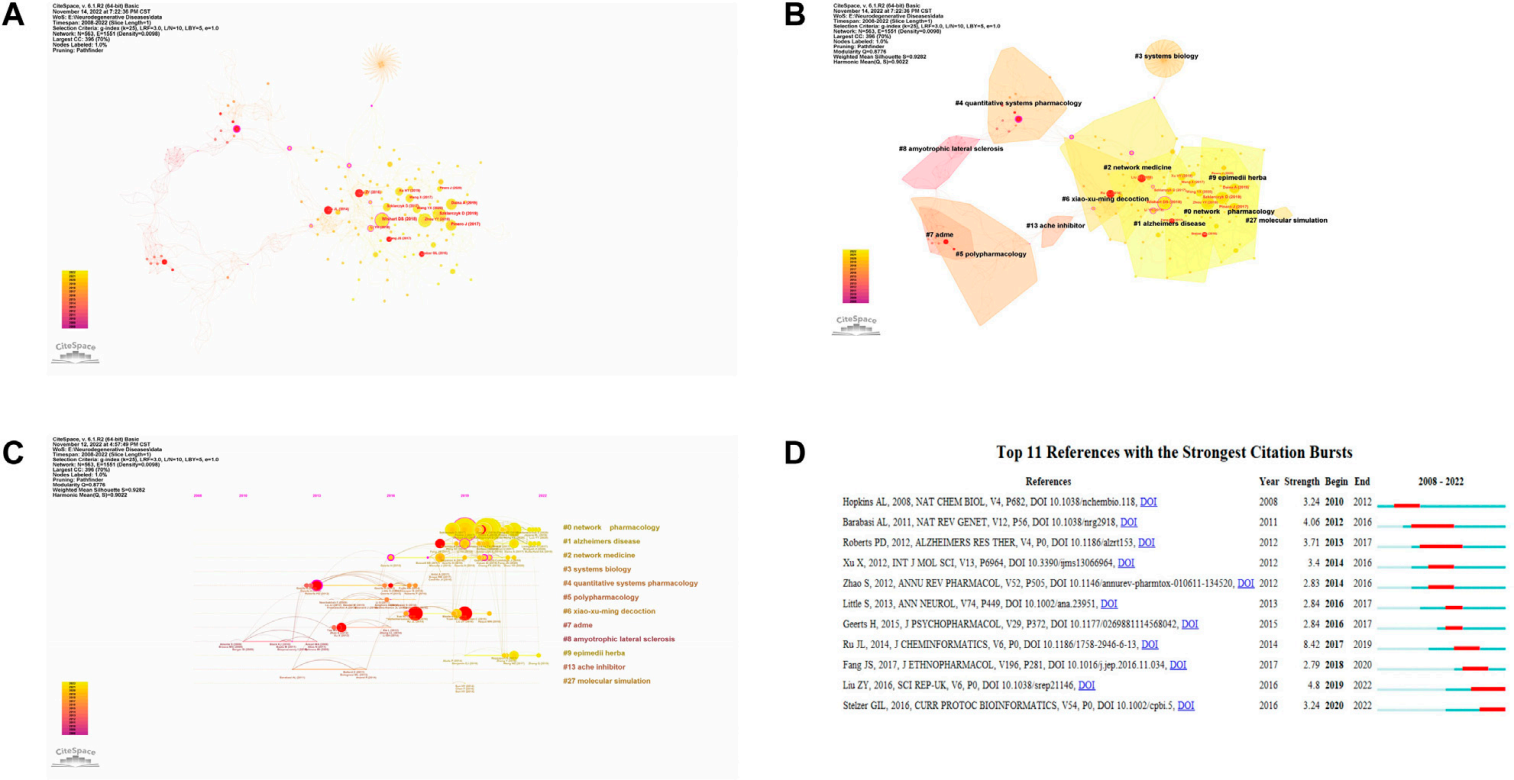
Analysis of most commonly cited references. **(A)** Visualization of co-cited references. **(B)** Cluster analysis of co-cited references. **(C)** Timeline graph of cluster analysis. **(D)** Top 11 references with the most strongest bursts.

Table 5 lists the highly cited articles, the top cited article was “TCMSP: a database of systems pharmacology for drug discovery from herbal medicines” by Ru JL in 2014 (94 citations), followed by Shannon P in 2003 (73 citations) and Hopkins AL in 2008 (68 citations). We built a visualization network of co-cited references (Figure 7A). “Wishart (2018),” “Szklarczyk D (2019),” and “Zhou YY (2019)” were frequently co-cited. Cluster analysis divided most of the relevant terms related to network pharmacology in NDs into 12 main categories (Table 6). The top 5 clusters were “network pharmacology,” “alzheimers disease,” “network medicine,” “systems biology,” and “quantitative systems pharmacology”. The silhouette value (S) represents the mean contour value of a cluster. Generally, if S > 0.5, cluster is considered proper, and if S > 0.7, cluster is persuasive. In this research, the S values of the top 12 clusters were more than 0.7, indicating that clustering was considered credible. Among all clusters, “network pharmacology” (Cluster 0#) was the largest cluster containing 68 articles (Table 6), which mainly discussed the application and the development of network pharmacology. In addition, references with the strong citations burst were mainly concentrated in the three clusters of 0#, 1#, 2# (Figure 7B), they also were the most recently formed clusters, suggesting that network pharmacology and alzheimers disease may be the latest hotspots.

**TABLE 5:** Top 10 cited literatures.

Rank	Citations^a^	Authors	Title	Source	Year	DOI
1	94	Ru JL	TCMSP: a database of systems pharmacology for drug discovery from herbal medicines	J CHEMINFORMATICS	2014	10.1186/1758-2946-6-13
2	73	Shannon P	Cytoscape: a software environment for integrated models of biomolecular interaction networks	GENOME RES	2003	10.1101/gr.1239303
3	68	Hopkins AL	Network pharmacology: the next paradigm in drug discovery	NAT CHEM BIOL	2008	10.1038/nchembio.118
4	40	Li S	Traditional Chinese medicine network pharmacology: theory, methodology and application	CHIN J NAT MEDICINES	2013	10.1016/S1875-5364 (13)60037-0
5	33	Hopkins AL	Network pharmacology	NAT CHEM BIOL	2007	10.1038/nbt1007-1110
6	31	Huang DW	Systematic and integrative analysis of large gene lists using DAVID bioinformatics resources	NAT PROTOC	2009	10.1038/nprot. 2008.211
7	29	Barabasi AL	Network medicine: a network-based approach to human disease	NAT REV GENET	2011	10.1038/nrg2918
8	29	Pinero J	DisGeNET: a comprehensive platform integrating information on human disease-associated genes and variants	NUCLEIC ACIDS RES	2017	10.1093/nar/gkw943
9	29	Wishart DS	DrugBank 5.0: a major update to the DrugBank database for 2018	NUCLEIC ACIDS RES	2018	10.1093/nar/gkx1037
10	69	Szklarczyk D	STRING v11: protein-protein association networks with increased coverage, supporting functional discovery in genome-wide experimental datasets	NUCLEIC ACIDS RES	2019	10.1093/nar/gky1131

^a^citations refers to the number of records in which this reference is cited in the obtained articles

**TABLE 6:** Top 12 largest clusters of co-cited references.

Cluster	Label (LLR^a^)	Size	Silhouette	Average year
#0	network pharmacology	68	0.923	2020
#1	alzheimers disease	55	0.833	2021
#2	network medicine	45	0.909	2020
#3	systems biology	44	1	2018
#4	quantitative systems pharmacology	44	0.938	2016
#5	polypharmacology	35	0.932	2015
#6	xiao-xu-ming decoction	25	0.915	2018
#7	adme	24	0.962	2014
#8	alzheimers disease	23	0.993	2011
#9	epimedii herba	19	0.921	2020
#13	ache inhibitor	10	0.996	2014
#27	molecular simulation	4	0.995	2019

^a^ LLR, log likelihood ratio.

Furthermore, we build a timeline for clusters in Figure 7C. Relatively, the application of network pharmacology and traditional Chinese medicine therapy of AD are newly concerned by researchers. Figure 7D lists the 11 references with the strongest citation explosions from 2010 to 2022. The most cited article was from Ru JL in 2014, which also had the largest number of citations. Based on the burst time, Barabasi AL in 2011 and Roberts PD in 2012 were most prominent. Barabasi AL et al. through the way of network pharmacology, systematically explored diseases’ pathogenic factors, recognition pathways and molecular relationships between different phenotypes to identify drug targets and biomarkers for specific diseases (Barabasi et al., 2011). Roberts PD et al. explored the potential of quantitative systems pharmacology in assessing drug activity and mechanism by analyzing the differential efficacy of memantine in different stages of Alzheimer’s disease (Roberts et al., 2012). The above results hinted that exploration and application of network pharmacology were widely concerned for a long time.

### Analysis of keywords

**FIGURE 8:**
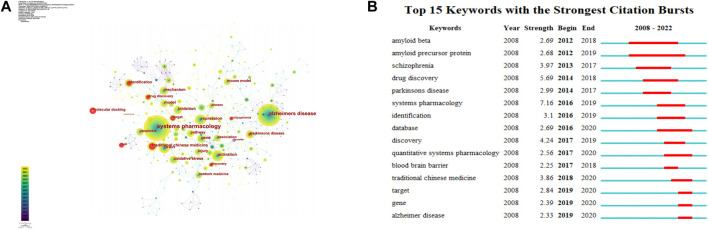
Analysis of keywords. **(A)** CiteSpace visualization of keywords. **(B)** Top 15 keywords with the most strongest bursts.

According to visual analysis of keywords by CiteSpace (Figure 8A), network pharmacology and Alzheimer’s disease were the two most prominent words. As shown in Table 7, the most common keywords were: network pharmacology, Alzheimer’s disease, brain, systems pharmacology and traditional Chinese medicine. Among them, double blind (0.24), inhibition (0.23), and amyloid precursor protein (0.2) showed highest intermediary centrality. The intermediary centrality represents the connectivity of a node, and the higher it is, the more terms can be connected through the node, indicating the importance of a node in the network.

**TABLE 7:** Top 10 keywords by frequency and centrality.

Rank	Frequency	Keyword	Centrality	Keyword
1	163	network pharmacology	0.24	double blind
2	112	alzheimers disease	0.23	inhibition
3	45	brain	0.20	amyloid precursor protein
4	42	systems pharmacology	0.19	mouse model
5	40	traditional chinese medicine	0.18	disease
6	39	activation	0.18	amyloid beta
7	38	expression	0.17	apoptosis
8	33	mechanism	0.17	schizophrenia
9	33	model	0.16	deep brain stimulation
10	32	inhibition	0.15	alzheimers disease

Burst keywords detection can reveal the evolution of the topic, predict the development directions and excavate potential flash point. As shown in Figure 7B, the keywords were mainly concentrated between 2012 and 2020. Besides, the burst keywords with strong strength were “systems pharmacology,” “drug discovery,” and “discovery”, indicating that the application of systems pharmacology and the search for drugs during this period may have been a major concern for researchers. Furthermore, “amyloid precursor protein” had been followed for the longest time, which was an important molecule in the pathogenesis of AD. In keyword burst detection, “systematic pharmacology” and “drug discovery” have highest intensity, “amyloid precursor protein” had longest sustained burst, and the latest bursts were concentrated in “systematic pharmacology,” “database,” “traditional Chinese medicine,” and “alzheimers disease” suggested that the current hotspots may be focused on the exploration of therapeutic methods for AD. Burst detection in this research contained major types, molecular mechanisms, and hot research methods of neurodegenerative diseases.

## Discussion

In this research, we utilized the method of bibliometrics to objectively analyze literatures allied to the network pharmacology in NDs. Our results suggested that the number of annual publications in this field had increased significantly in recent years, which showed that this field was receiving more and more attention. The number of publications and citations in a certain research field are considered to be significant index to evaluate academic reputation and scientific research ability of a country or institution (Soteriades and Falagas, 2005; Yadava et al., 2019). China ranked first in research in terms of publications, but average citation was not high, suggesting the quality of researches led by China was not satisfactory. As for institutes, most of the top 10 institutions were from China, but the cooperation between institutions was not strong. Beijing Univ Chinese Med has the most publications and Chinese Acad Sci ranked first in terms of TGCS. As the second prolific country, the United States had close cooperation with other countries, as well as the highest centrality in the cooperative network, representing high quality of its researches and far-reaching effects on other countries. Transnational cooperation has significant benefits in thinking innovation and model reform, becoming an important trend in future research (Gal et al., 2017). The above results indicated that Chinese institutions were dominant in this field, while United States had the best cooperation with other countries.

In addition, our results indicated that Geerts H was the most influential author. He focused on Alzheimer’s drug research and made important contributions to the treatment of NDs. He devoted himself to using systems pharmacology to develop integrated models of compounds, mechanisms, and disease-level data (Geerts et al., 2020), explored intelligent and experience-based computer model (Geerts et al., 2016). The second most productive author was Fang JS from China, who was devoted to multiple studies of TCM and drug discovery in NDs. He proposed using network pharmacology to explore the potential mechanisms of the most widely used medicinal herbs of AD (Fang et al., 2017a). Besides, he established a predictive knowledge base (AlzhCPI) of chemical-protein interactions in AD (Fang et al., 2015), as well as a genome-wide positioning systems platform (AlzGPS) for AD drug discovery (Zhou et al., 2021), and constructed a worldwide drug-target network of natural products by integrating predicted drug-target interactions (DTI) (Fang et al., 2017b). Recently, he also proposed endophenotype-based network medicine approach to promote AD therapeutic development (Fang et al., 2020). Simultaneously, the most co-citations author was Hopkins AL. He proposed “Network pharmacology”, which was a new framework for thinking about how to innovate drug by understanding the biological and kinetic properties of drugs to improve clinical efficacy and recognize side effects and toxicities (Hopkins, 2008). The above authors enjoyed a high academic reputations and had made a significant contribution to the developments and progress in the field relative to network pharmacology in NDs.

In this research, most prolific journals were divided into Q1 or Q2, and Alzheimer & Dementia (IF16.655) had the highest IF. Frontiers in Pharmacology (IF5.998) were the journal with top production, noting that many high-quality articles had been published about this filed. Although Evidence-based Complimentary and Alternative Medicine (IF2.650) had 33 publications, its IF values were less than 5. Maintaining the quality of the work as well as increasing the output will help enhancing the academic impact of journal. Among the most commonly cited journals, Nature (IF69.504), Science (IF63.714) and Nucleic Acids Research (IF19.160) had IF values higher than 15, indicating that the quality of the evidences from the above periodicals was high, and had strong persuasiveness and influence.

The analysis of co-cited articles and literature cluster reflect the core content and hot topics in a field, and the highly cited documents can prompt the core research and high-intensity burst can represent emerging academic hotspots. (Chen et al., 2022). Top three articles with highest citations are as follow: ⅰ) Ru JL in 2014 (94 citations), which also has strongest burst intensity. This research established a traditional Chinese medicine systems pharmacology database and analysis platform (TCMSP) based on Chinese herbal Systems pharmacology, integrating the chemical properties, targets, related diseases and interaction networks of Chinese herbal medicines (Ru et al., 2014). It is manifested that the research and development of Chinese herbal medicine employing systems pharmacology may be a new hotspot in current period. ⅱ) Shannon P in 2003 (73 citations) developed a generic model for biomolecular interaction networks and states: Cytoscape (Shannon et al., 2003). ⅲ) Hopkins AL in 2008 (68 citations) reported that based on drug, disease, gene and target interaction network, network pharmacology can systematically and comprehensively understand the drugs on the body’s disease network intervention and impact, guiding the discovery of new drugs and new targets (Hopkins, 2008). Briefly, the highly cited articles are all new explorations based on databases and interactive networks, indicating that network pharmacology is being widely applied to the research of molecules and diseases and the development of drugs.

Additionally, “network pharmacology” (Cluster ID 0#) with 68 references was the largest cluster, which covered network pharmacology in the applications of drug discovery, protein, gene etc. Peng Zeng et al. revealed the key components in Ginkgo Folium and its mechanism in the treatment of AD employing network pharmacology (Zeng et al., 2021). Szklarczyk et al. established a database of high-throughput text mining and internet clustering by collecting information of protein-protein interaction (Szklarczyk et al., 2019). Pinerol J et al. developed DisGeNET, integrating human disease-related genes, scientific literature data, animal model studies, and the GWAS catalog, which play an significant role in the research of molecular basis in disease and its side effects, characteristics of disease genes, drug therapeutic effects and adverse reactions, validation of gene prediction of disease and evaluation of text mining methods (Pinero et al., 2017). Moreover, the latest cluster includes “alzheimers disease” (Cluster ID 1#), “network pharmacology” (Cluster ID 0#), “network medicine” (Cluster ID 2#) and “epimedii herba” (Cluster ID 9#). It was found that multiple clustering topics were related to network pharmacology, which also had high frequency in the keyword co-occurrence network, indicating that network pharmacology is the research core in this field. Finally, the article with the highest centrality is Geert H in 2020, which explored new approaches for molecular, target recognition, therapeutic drug discovery and development in central nervous system based on quantitative systems pharmacology (QSP) (Geerts et al., 2020). In conclusion, based on the analysis of literature clustering and co-citation, network pharmacology has recently emerged as the flash point of this filed, and has received extensive concern from scholars around the world.

Based on the joint analysis of keywords’ frequency and emergence, the study on therapy for AD especially TCM draw extensive attention recently. These TCM therapy of AD included the follow aspects: 1) reducing the deposition of Aβ peptide and removing the hyperphosphorylation TAU protein: gypenosides XVII enhanced the autophagy dependent removal of Aβ through TFEB activation (Meng et al., 2016). Geniposide decreased amyloid deposition and promoted autophagy by modulating mTOR signal pathway (Zhang et al., 2019). Resveratrol by means of modulating GSK-3β, CaMKII, and PP2A to reduce TAU deposition (He et al., 2016). 2) regulating cholinergic neurotransmitters: huperzine A was proven to be a powerful neuroprotective agent in inhibiting robust anti-acetylcholinesterase (ACHE) activity (Friedli and Inestrosa, 2021). 3) improving blood vessel and microcirculation: combination therapy of Buyang Huanwu Tang and MSCs transplantation may repair vascular injury by up-regulating the expression of VEGF and Ki-67 (Zhang et al., 2010). 4) reducing oxidative stress and inflammation: Danggui-Shaoyao-San can improve the apoptosis of brain neurons induced by oxidative stress (Lan et al., 2012), Ginkgolide B (GB) inhibits NLRP3 inflammasome activation and promotes microglia M2 polarization to reduce neuroinflammation (Zeng et al., 2021). Crocin can attenuate malathion-induced changes and cognitive impairment in the nervous system by reducing oxidative stress and inflammation (Mohammadzadeh et al., 2020). 5) anti-apoptotic effect of neurons: Ginsenoside RG2 can protect PC12 cells from Aβ25-35-induced apoptosis by enhancing PI3K/Akt signaling pathway (Cui et al., 2017). Above all, plenty of evidences have proved that the natural extract of TCM has great potential in the treatment of AD by multiple pathways and targets (Jarrell et al., 2018). Network pharmacology is a new approach to the study of drugs, diseases, and targets in TCM, which mainly contains: 1) exploring the mechanisms of Chinese herbal formulae: Qu et al. analyzed the antidepressant activity of Huang-Lian Jie-Du Decoction (HLJDD) employing network pharmacology and metabolomics, and found that SLC6A4 and MAOA in tryptophan metabolism may be the major antidepressant targets of HLJDD (Qu et al., 2021). 2) identifying the active ingredients of Chinese herbal compound: Wu et al. analyzed the composition and targets of Citri Reticulatae Pericarpium based on systems pharmacology methods and vitro experiments, and validated potential molecular mechanisms in the treatment of liver injury (Wu et al., 2021). Taken together, network pharmacology in TCM therapy especially for AD may become a new focus and direction of academic research in the future.

### Limitation

This study visualized the relationships between network pharmacology and NDs to understand their trends and hotspots through the use of bibliometrics. However, there are a few shortcomings to this study. For instance, due to the limitations of current scientific measurement software, it is difficult to analyze data from multiple databases simultaneously, so this study only utilized the WOSCC database. Moreover, this study only included articles and reviews, not abstracts, conferences, or books. Therefore, more comprehensive studies and databases could be undertaken in the future for more complete and accurate analysis.

## Conclusion

In conclusion, researches on NDs employing network pharmacology have been extensively conducted, and the number of articles retrieved has shown a clear increasing trend, especially in 2014–2022. China was the most prolific country, while Chinese Academy of Sciences also made important research achievements, which was important force in the development of this field. Moreover, Geerts H was the leader in this field by publishing the most articles. The main research focuses in this area are the development and application of network pharmacology and the treatment of AD. Medication of NDs, especially TCM, will become the focus of future research. In addition, the application of network pharmacology in Chinese herbal compound have great research prospects. Collectively, our study conducted a systematic visualization of literatures on network pharmacology in NDs, showed the main aspects of the subject and provides the direction and reference for further researches into diseases and drugs.

## Data Availability

The original contributions presented in the study are included in the article/supplementary material, further inquiries can be directed to the corresponding authors.
